# Recurrent Drought Conditions Enhance the Induction of Drought Stress Memory Genes in *Glycine max* L.

**DOI:** 10.3389/fgene.2020.576086

**Published:** 2020-10-09

**Authors:** Yeon-Ki Kim, Songhwa Chae, Nam-Iee Oh, Nguyen Hoai Nguyen, Jong-Joo Cheong

**Affiliations:** ^1^Department of Biosciences and Bioinformatics, Myongji University, Yongin, South Korea; ^2^Center for Food and Bioconvergence, Seoul National University, Seoul, South Korea

**Keywords:** DNA chip, drought, stress memory, gene ontology, transcript, microarray, soybean

## Abstract

Plants remember what they have experienced and are thereby able to confront repeated stresses more promptly and strongly. A subset of the drought responsive genes, called stress memory genes, displayed greatly elevated levels under recurrent drought conditions. To screen for a set of drought stress memory genes in soybean (*Glycine max* L.), we designed a 180K DNA chip comprising 60-bp probes synthesized *in situ* to examine 55,589 loci. Through microarray analysis using the DNA chip, we identified 2,162 and 2,385 genes with more than fourfold increases or decreases in transcript levels, respectively, under initial (first) drought stress conditions, when compared with the non-treated control. The transcript levels of the drought-responsive genes returned to basal levels during recovery (watered) states, and 392 and 613 genes displayed more than fourfold elevated or reduced levels, respectively, under subsequent (second) drought conditions, when compared to those observed under the first drought stress conditions. Gene Ontology and MapMan analyses classified the drought-induced memory genes exhibiting elevated levels of transcripts into several functional categories, including those involved in tolerance responses to abiotic stresses, which encode transcription factors, protein phosphatase 2Cs, and late embryogenesis abundant proteins. The drought-repressed memory genes exhibiting reduced levels of transcripts were classified into categories including photosynthesis and primary metabolism. Co-expression network analysis revealed that the soybean drought-induced and -repressed memory genes were equivalent to 172 and 311 Arabidopsis genes, respectively. The soybean drought stress memory genes include genes involved in the dehydration memory responses of Arabidopsis.

## Introduction

Prolonged or habitual drought may be one of the most serious detrimental stresses for crops during their lifetime. Plants have evolved various strategies to cope with such drought conditions by exhibiting many physiological and developmental changes through regulation of gene expression (Shinozaki and Yamaguchi-Shinozaki, 1996; Shanker et al., 2014). Under drought conditions, many drought-responsive genes are expressed, and their transcript levels are returned to basal levels during recovery (watered) states. Thus, during recurring cycles of dehydration stresses, their transcript levels rise and fall repeatedly.

Plants can control their responses to repeated stress by altering the expression patterns of the responsive genes. A subset of genes called ‘memory genes’ are expressed at highly elevated or reduced levels during subsequent dehydration events (Ding et al., 2012; Kinoshita and Seki, 2014; Godwin and Farrona, 2020), thereby enabling plants to respond more promptly and strongly to the repeated drought stress. This response has been referred to as memory, imprinting, priming, training, and acclimation to stress (Bruce et al., 2007; Conrath, 2011; Walter et al., 2011). For example, Ding et al. (2012) showed that Arabidopsis plants trained with previous dehydration events wilted much slower than non-trained plants.

The potential of stress memory for enhancing crop productivity under drought conditions has been explored. In particular, Ramírez et al. (2015) improved potato drought tolerance through the induction of long-term water stress memory. Wang et al. (2014) improved tolerance to drought stress by priming before anthesis in wheat. Abdallah et al. (2017) performed drought priming to improve the productivity of olive under severe drought conditions. In addition, Tabassum et al. (2018) showed that terminal drought and seed priming improved drought tolerance in bread wheat.

The molecular mechanisms underlying stress memory are not yet fully understood. The most plausible mechanism involves epigenetic changes in the chromatin architecture of memory genes, such as promoter DNA methylation, histone modifications, or small RNA generation (Luo et al., 2012; Avramova, 2015; Kim et al., 2015; Lämke and Bäurle, 2017; Chang et al., 2019). Such an epigenetically modified status is transmitted mitotically to newly developed cells during the cell division process.

Furthermore, the epigenetic status of memory could be transmitted meiotically to the next generation of plants, exhibiting transgenerational epigenetic inheritance (Molinier et al., 2006; Boyko and Kovalchuk, 2011; Quadrana and Colot, 2016). The potential of transgenerational inheritance of acquired traits has been applied to improve crop productivity (Springer, 2013; Mickelbart et al., 2015; Bilichak and Kovalchuk, 2016). For instance, Verkest et al. (2015) improved drought tolerance in canola epi-lines by repeatedly selecting for increased drought tolerance for three generations. Tabassum et al. (2017) reported that seed priming and transgenerational drought memory improved tolerance against drought and salt stress in bread wheat. In addition, Raju et al. (2018) developed an epigenetic breeding system in soybean for increased yield and stability, by modulation of development, defense, phytohormone and abiotic stress response pathways.

The first step toward understanding the molecular mechanism of transcriptional memory and its wide application in crop improvement is the identification of stress memory genes. Many dehydration stress memory genes have been identified in Arabidopsis (Ding et al., 2013), *Zea mays* (Ding et al., 2014; Virlouvet et al., 2018), rice (Li et al., 2019), and potato (Chen et al., 2020). In this study, we identified drought stress memory genes in soybean by microarray analysis using a newly designed 180K DNA chip.

## Materials and Methods

### Plant Materials and Treatments

Certified quality soybean seed (*Glycine max* L. cv. Daepoong) was obtained from the Korea Seed and Variety Service. The seeds were sown in potting soil at 15 mm depth in 50-well plates, with one seed per well (35 mm width × 35 mm length × 45 mm depth), and incubated in a growth chamber at 28°C and 50–60% relative humidity under a 16-h light (8,000 lux)/8-h dark photoperiod (Supplementary Figure 1). Water was supplied from a watering tray located beneath the plate. After growth for 7 days, when the primary leaves of soybean plantlets were fully open, one of the two plates was exposed to drought stress by withdrawing the watering tray for 4 days. Eight to twelve primary leaves were collected from the watered control (WT1) and drought-treated (DR1) plates, and frozen in liquid nitrogen. Then, the (first) drought-treated plate was re-watered for 1 day (WT2), and the second drought treatment was conducted for 4 days (DR2). This experiment was performed twice independently.

### DNA Chip Design

Based on the whole soybean (*G. max* var. Williams 82) genome database available from Phytozome^1^ (Goodstein et al., 2012), three 60-nt-long feature probes were designed based on a representative transcript of each gene, beginning 60 bp upstream from the end of the stop codon and shifting downstream at 30-bp intervals; the three resulting probes covered 120 bp: 60 bp of coding sequence (CDS) and 60 bp in the 3′ untranslated region (3′-UTR). Additional probes for alternatively spliced transcripts, chloroplast genes, mitochondrial genes, and selection markers, such as *gfp*, *gus*, *hyg*, *bar*, and *kan*, were included. A total of 175,391 probes were designed and synthesized *in situ* on the 180K DNA chip, which was manufactured by Agilent Technologies (Santa Clara, CA, United States).

### Microarray Analysis

Two independent biological replicates of microarray experiments were performed using soybean plants treated with or without water deprivation. Total RNA was isolated from soybean leaves as described previously (Chae et al., 2017), and probes were labeled using the Low RNA Input Linear Amplification Kit PLUS (Agilent Technologies), following the manufacturer’s protocol^2^. Total RNA (500 ng) was used to generate labeled cRNA by incorporating cyanine CTP. The microarray was scanned using a DNA microarray scanner (G2505C; Agilent Technologies) and raw intensity data were extracted using the feature extraction software. Background adjustment and normalization of microarray the data were achieved using the Limma package in the R computing environment^3^. The distribution of probe intensities from eight microarrays was analyzed using the plotDensities function. Frequency densities before and after normalization were plotted against log2-based intensities. Genes exhibiting > 4-fold enhanced transcription levels in two independent experiments were considered to show significantly increased expression. Expression profiling was conducted with the *Glycine max* Alternatively Spliced Transcript Detecting Microarray (GmASDM).

### Bioinformatics Analysis

Multiple analyses were performed using Limma (Chae et al., 2016), which adopts the linear modeling approach implemented by lmFit and the empirical Bayesian statistics implemented by eBayes. Genes with an adjusted *P*-value < 0.05 were collected and further assessed in terns of whether they had expression levels > 1 or < -1 for at least one stage compared to the control.

Biological term enrichment among genes was assessed using GoMiner (Ashburner et al., 2000; Zeeberg et al., 2003). To identify a tentative ortholog of a *G. max* gene in the Arabidopsis genome, BLASTP analysis was performed for the two species; genes with a score ≥ 70 were tentatively considered as counterparts. The microarray contained 68,234 transcripts/54,076 genes, 19,750 of which matched Arabidopsis genes with scores ≥ 70; these genes were used as the total gene set in GoMiner. Terms with significant changes in the biological processes category were collected and subjected to hierarchical clustering using the Limma language of the Bioconductor project^4^. False discovery rate (FDR) values were obtained from 100 randomizations. Gene Ontology (GO) terms with FDR values < 0.05 in at least one group were collected. To present GO terms in hierarchical clusters, unique GO terms were first selected. FDR values < 0.05 were scaled from 0.5 to 5, such that the more enriched terms had values closer to 5 and a darker color on the heatmap(see footnote 4). Other non-significant FDR values were transformed to 0 using the Perl scripting language. To reduce redundancy, the top terms in each clade were selected and hierarchical clustering was performed with the Bioconductor R package.

To determine the enriched pathways, MapMan (ver. 3.6) analysis was conducted using AGI_TAIR9 as the mapping database. Pathways were generated with the highest homolog of Arabidopsis genes extracted from soybean gene transcripts, as described above.

Co-expression networks were established for the soybean transcripts using ArabidopsisNet^5^. The database was constructed using 994 microarrays from the Arabidopsis ATH1 Genome Array (GPL198), downloaded from the Gene Expression Omnibus (GEO^6^). The co-expression database was constructed based on Pearson coefficients, as described for RiceArrayNet (Lee et al., 2009) and RapaNet (Kim et al., 2017). To test the clustered genes, correlation information was generated in the Newick format and transformed into network and tree diagrams using the Molecular Evolutionary Genetics Analysis program (MEGA X; Kumar et al., 2018).

### Quantitative Reverse-Transcription Polymerase Chain Reaction (qRT-PCR)

Total RNA was extracted from soybean leaves using the Spectrum Plant Total RNA kit (Sigma-Aldrich, St. Louis, MO, United States). First-strand cDNA was synthesized using the SuperScript III First-Strand Synthesis SuperMix for qRT-PCR (Invitrogen, Carlsbad, CA, United States). Quantitative PCR (qPCR) was carried out using SolGent 2 × Real-Time Smart Mix (SolGent, Daejeon, South Korea) with specific primers. Thermocycling and fluorescence detection were performed using the Mx3005P qPCR system (Agilent Technologies). The PCR program was as follows: 95°C for 15 min, followed by 40 cycles of 95°C for 30 s, 60°C for 30 s, and 72°C for 30 s. Soybean 60S rDNA was used as an internal control. The qRT-PCR experiments were repeated independently three times. Statistical analysis was performed using Duncan’s test (Duncan, 1955) at a 95% confidence level.

## Results

### DNA Chip Design

The whole soybean (*G. max* var. Williams 82) genome has been sequenced (Schmutz et al., 2010), and the database is available through Phytozome^7^ (Goodstein et al., 2012). In total, 87,977 transcripts from 55,589 loci have been annotated at *Glycine max* Wm82.a2.v1^8^. Among these, single transcripts are expressed from 40,372 loci, whereas 47,605 transcripts are from 15,217 loci with alternative splicing (Supplementary Table 1A). We designed a microarray to contain 40,261 single transcripts and 27,873 alternative transcripts. In total, 68,234 transcripts were discerned among the 87,977 annotated transcripts.

In most cases, three 60-nt-long probes were designed from a representative transcript of each gene, starting 60 bp upstream of the stop codon and shifting downstream at 30-bp intervals (Supplementary Figure 2A). Thus, the three probes covered 120 bp: 60 bp of the CDS and 60 bp in the 3′-UTR of each transcript. Due to overlapping sequences, only two probes were designed for certain genes to ensure specificity. For some genes, a representative transcript is distinguished by a unique exon (UE) among the alternative spliced transcripts, and an additional probe was designed. In addition, 83 chloroplast and 88 mitochondrial genes (V1_YP) were included in the DNA chip. Several election markers such as *gfp*, *gus*, *hyg*, *bar*, and *kan* were also included as negative controls, to cover 68,954 transcripts. In total, 175,391 probes were designed and synthesized *in situ* on the 180K DNA chip.

### Drought Stress Memory Genes in Soybean

To test for genes involved in the recurring water stress, plants were exposed to cycles of watering and water deprivation. The seedlings were grown for 7 days, then exposed to drought stress for 4 days by removal of the water tray (Supplementary Figure 1). The first leaves of well-watered (WT1) and drought-treated (DR1) soybeans were collected and used for RNA extraction (Supplementary Figure 2B), and transcript levels of drought-responsive genes were analyzed with the 180K soybean DNA chip.

Microarray data were collected from biological replicates and the consistency between samples was tested (Supplementary Figure 3A). Log_2_-based intensities of two WT1 samples were compared, and the linear model *y* = 0.98*x* + 0.018 was obtained, with a Pearson correlation coefficient of 0.97. The data were corrected for background and normalized using the Limma package (Supplementary Figures 3B,C), as described in the Methods section. Signal intensities after normalization are presented in Supplementary Table 2A.

The median intensity of sample WT1 was 231 and its maximum value was 535,859.7. The intensities of photosynthesis genes such as Chloroa_b-bind, RuBisCO_small subunit, and RbcS protein ranged from 366,252 to 393,034. In the first drought stress treatment (DR1), the median intensity of sample DR1 was 232.7 and its maximum value was 538,439.3. The intensities of genes such as Metallothio_2 family, PsbR family, ABA_WDS family, and RuBisCO_small domain proteins ranged from 296,733.2 to 369,185.6. Through the repeated watering treatments, photosynthesis genes such as RuBisCO_small subunit, Chloroa_b-bind, and RbcS proteins returned to their maximum induction levels. Meanwhile, through the recurring drought stress treatments, Dehydrin family, Metallothio_2 family, Tryp_alpha_amyl family, and ABA_WDS family reached the maximum expression levels among the highly induced genes.

From these normalized data, we classified the primary drought-responsive transcripts as those that were induced or repressed at the first drought treatment (DR1). Approximately 88% (60,386) of the 68,954 examined transcripts were detected in the microarray analysis (Table 1 and Supplementary Table 2A). Compared to the well-watered control (WT1), 2,162 transcripts showed 4-fold increases (Table 1 and Supplementary Tables 1B, 2B), whereas 2,385 transcripts showed 4-fold decreases (Supplementary Table 1B, 2C). These were categorized as drought-repressed transcripts (DRTs) and drought-induced transcripts (DITs), respectively.

**TABLE 1:** Gene Ontology terms enriched with DRTs, DITs, DRMTs, and DIMTs.^*a*^

**Gene Ontology terms (GO_id)**	**DRT**	**DIT**	**DRMT**	**DIMT**
GO:0005992_trehalose biosynthetic process	0	0	0	1.58
GO:0009409_response to cold	0	5	0	5
GO:0009611_response to wounding	0	5	0	1.58
GO:0009788_negative regulation of ABA-activated signaling	0	1.04	0	1.31
GO:0009873_ethylene-activated signaling pathway	0	5	0	5
GO:0010200_response to chitin	0	1.85	0	5
GO:0010286_heat acclimation	0	5	0	5
GO:0042538_hyperosmotic salinity response	0	5	0	5
GO:0006364_rRNA processing	−1.37	0	−5	0
GO:0006636_unsaturated fatty acid biosynthetic process	−5	0	−5	0
GO:0009637_response to blue light	−5	0	−5	0
GO:0009765_photosynthesis, light harvesting	−5	0	−5	0
GO:0009773_photosynthetic electron transport in photosystem I	−5	0	−5	0
GO:0010075_regulation of meristem growth	−5	0	−5	0
GO:0010103_stomatal complex morphogenesis	−5	0	−5	0
GO:0010114_response to red light	−5	0	−5	0
GO:0010155_regulation of proton transport	−5	0	−5	0
GO:0010207_photosystem II assembly	−1.65	0	−5	0
GO:0010218_response to far red light	−5	0	−5	0
GO:0015995_chlorophyll biosynthetic process	−5	0	−5	0
GO:0016117_carotenoid biosynthetic process	−1.04	0	−5	0
GO:0016556_mRNA modification	−1.05	0	−5	0
GO:0018298_protein-chromophore linkage	−1.55	0	−5	0
GO:0019288_methylerythritol 4-phosphate pathway	−5	0	−5	0
GO:0019344_cysteine biosynthetic process	−5	0	−5	0
GO:0034660_ncRNA metabolic process	0	0	−5	0
GO:0042254_ribosome biogenesis	0	0	−5	0

*^*a*^Selected from **Supplementary Table 3A**.*

When the DR1 plants were re-watered for 1 day (WT2), transcript levels recovered to those of non-stressed (WT1) plants. As the second drought stress proceeded for 4 days (DR2), transcript levels of the drought-responsive genes increased or decreased to the levels detected in the DR1 plants (Supplementary Table 2B,C). However, among them, transcript levels of 613 genes decreased 4-fold, while 392 genes increased 4-fold (Supplementary Tables 1B, 2D,E) under the second drought conditions (DR2), when compared to those observed under the first drought stress (DR1) conditions. We hypothesized that the drought-induced expression of these genes was influenced by the previous drought stress (DR1), and thus these genes were designated drought-induced memory transcripts (DIMTs) or drought-repressed memory transcripts (DRMTs), respectively. It is notable that no memory transcript was identified among the chloroplast or mitochondrial genes (Supplementary Table 1B).

The microarray datasets have been deposited at the National Center for Biotechnology Information (NCBI) GEO database (see footnote 6) under GEO accession number GSE153660.

### GO and MapMan Analyses

To test for enriched GO terms, the DITs, DRTs, DIMTs, and DRMTs were subjected to GO analysis (Supplementary Figure 4). The biological function of each gene was inferred based on the conserved sequence motifs (domain) contained in the encoded protein and the most homologous Arabidopsis gene. The terms enriched among the DITs and DIMTs included the trehalose biosynthetic process, response to cold, response to wounding, negative regulation of abscisic acid (ABA)-activated signaling, ethylene-activated signaling pathway response to chitin, heat acclimation, and hyperosmotic salinity response were highly enriched (Table 1 and Supplementary Table 3A). In addition, genes encoding protein phosphatase 2C (PP2C) family proteins and late embryogenesis abundant (LEA) proteins were also included in this group. By contrast, DRTs and DRMTs involved in the chlorophyll biosynthetic process, photosynthesis, light harvesting, and response to red or blue light was significantly repressed (Table 1 and Supplementary Table 3A).

**FIGURE 1:**
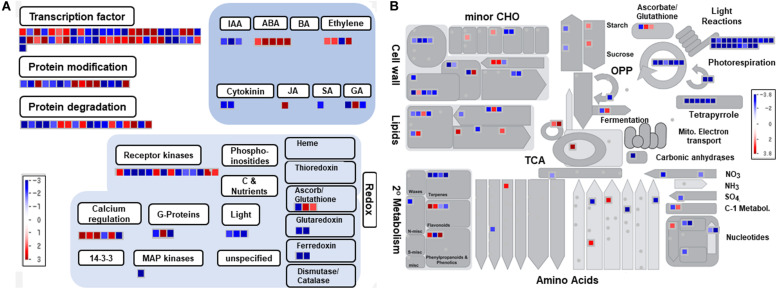
Functional overview of soybean drought-induced and drought-repressed memory gene transcripts. **(A)** Regulation overview. **(B)** Metabolism overview. The MapMan program (ver. 3.6) was used along with AGI_TAIR9 as the mapping database. Red and blue spots represent drought-induced memory and drought-repressed memory signals, respectively.

The MapMan analysis revealed that 190 gene transcripts out of 392 DIMTs have Arabidopsis equivalents functioning in the hormonal and transcriptional regulation in abiotic/biotic stress responses (Supplementary Table 3B). A number of ABA- and ethylene-responsive genes encoding a putative ABA deficient 2 (Glyma.11G180800-1), ABA 8′-hydroxylase (Glyma.16G109300-1), ABA-responsive protein-related (Glyma.19G100000-1), osmotin 34 (Glyma.11G025600-1), and ethylene responsive factor 1 (Glyma.10G007000-1) were highly upregulated under the second drought conditions (Figure 1A). Genes involved in ABA and ethylene synthesis were also induced, as shown by Glyma.08G176300-1 (9-*cis*-epoxycarotenoid dioxygenase), Glyma.08G255400-1 (9-*cis*-epoxycarotenoid dioxygenase), and Glyma.02G084400-1 (2-oxoglutarate-dependent dioxygenase). Among the biotic stress genes, the expression levels of several genes encoding disease resistance proteins, such as TIR-NBS-LRR class (Glyma.11G153000-1 and Glyma.16G214200-1), CC-NBS-LRR class (Glyma.05G082200-2), and downy mildew resistant 6 (Glyma.18G273200-1), were also increased. Genes encoding proteins involved in secondary metabolism, such as phenylpropanoid transferase family protein (Glyma.04G040000-1) and mannitol dehydrogenase (Glyma.07G175200-1, Glyma.16G096300-1, Glyma.01G021000-1, and Glyma.08G280500-1), were also included in the DIMTs.

Among 613 DRMTs, 349 genes have equivalents in Arabidopsis (Supplementary Table 3C). The metabolism overview showed that photosynthesis was severely affected, with significantly reduced gene transcript levels (Figure 1B). Those genes encode a putative chloroplast thylakoid membrane protein (Glyma.07G019400-1), light harvesting complex PSII subunit 6 (Glyma.05G119000-1 and Glyma.08G074000-1), cytochrome b6f complex subunit (Glyma.07G163600-1, Glyma.20G008600-1, and Glyma.07G163600-1), chlorophyll binding (Glyma.16G162600-1, Glyma.16G165200-1, Glyma.16G165800-1, and Glyma.16G165500-1), photosystem II light harvesting complex protein 2.1 (Glyma.02G305400-1), and ATPC1 (Glyma.13G204800-2). In addition, genes encoding proteins involved in primary metabolic pathways, such as oxidoreductase (Glyma.12G222200-1, Glyma.12G150400-1, Glyma.12G222200-1, and Glyma.06G247100-1), tetrapyrrole binding (Glyma.12G052500-1 and Glyma.11G127800-1), and glutamyl-tRNA reductase (Glyma.02G218300-1 and Glyma.14G185700-1), were repressed. Hormone metabolism was also affected by cytochrome b561/ferric reductase (Glyma.07G055800-1), and auxin-responsive family protein (Glyma.02G049200-1), GAST1 protein homolog 4 (Glyma.09G238300-1), and gibberellin 2-beta-dioxygenase (Glyma.11G003200-1) expression levels were decreased. Protein degradation processes mediated by serine-type endopeptidase (Glyma.05G152200-1), subtilase family protein (Glyma.16G018900-1 and Glyma.07G050200-1), and serine-type endopeptidase (Glyma.04G044600-1, Glyma.06G045100-1, Glyma.14G087500-1, and Glyma.17G236800-1) were repressed.

### Co-expression Networks

**FIGURE 2:**
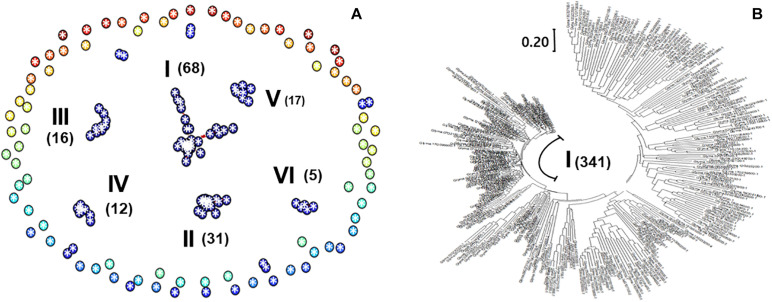
Co-expression networks. The criteria for a depth search of 0 with an absolute correlation value of 0.65 were applied in ArabidopsisNet (http://bioinfo.mju.ac.kr/arraynet/Arabidopsis). **(A)** Network diagram for soybean drought-induced memory genes. Circles represent soybean genes equivalent to Arabidopsis genes. The 392 soybean transcripts equivalent to 172 Arabidopsis genes form 11 groups, including six major clusters (I–VI). Numbers of associated members are presented in the parentheses. **(B)** A tree diagram of soybean drought-repressed gene transcripts. A tree consisting of 102 clusters was produced for 613 soybean drought-repressed gene transcripts. The Newick format was first employed with 349 Arabidopsis equivalent genes; then, the genes were replaced with representative soybean transcripts. The tree was drawn with the MEGA X program (Kumar et al., 2018).

The 392 transcripts in the DIMT group were equivalent to 172 Arabidopsis genes that constitute 11 groups including 6 major clusters, as shown in the network diagram (Figure 2A). The largest group, cluster I, comprises 68 soybean gene transcripts equivalent to 34 Arabidopsis genes (Supplementary Table 3D). This group of genes includes Glyma.19G147200-1 (LEA group 1 domain-containing protein), Glyma.10G277800-1 (Myb family transcription factor), Glyma.19G009100-1 (formate dehydrogenase), Glyma.01G195500-2 (ACT domain repeat 1), Glyma.06G177800-1 (C2 domain-containing protein/GRAM domain-containing protein), and Glyma.12G088300-1 (NAD^+^ ADP-ribosyltransferase). The second largest group (cluster II) was composed of 31 soybean gene transcripts equivalent to 17 Arabidopsis members, including Glyma.08G178100-1 (2-nitropropane dioxygenase family/NPD family), Glyma.16G203500-1 (C_3_HC_4_-type ring finger protein), Glyma.19G137400-1 (transferase, transferring glycosyl groups), Glyma.11G242900-1 (epsin N-terminal homology domain-containing protein/clathrin assembly protein-related), and Glyma.01G146500-1 (protein binding/ubiquitin-protein ligase/zinc ion binding).

Using the 613 DRMTs, a tree diagram consisting of 102 clusters was produced with 311 Arabidopsis equivalents (Figure 2B). The first major group was composed of 341 transcripts that equivalent to 203 Arabidopsis genes (Supplementary Table 3E), including those encoding cytochrome b6f complex subunit (Glyma.07G163600-1, Glyma.20G008600-1, and Glyma.07G163600-1), chlorophyllide a oxygenase (Glyma.14G150600-1), and photosystem II reaction center PsbP family protein (Glyma.03G230300-1 and Glyma.19G227400-1).

### qRT-PCR Analysis

We randomly selected eight DIMT genes that appeared to be involved in drought memory response and performed qRT-PCR analysis to confirm the microarray data. For each gene, a set of specific PCR primers was designed (Supplementary Figure 5), and their sequence specificity was examined by PCR with soybean genomic DNA.

**FIGURE 3:**
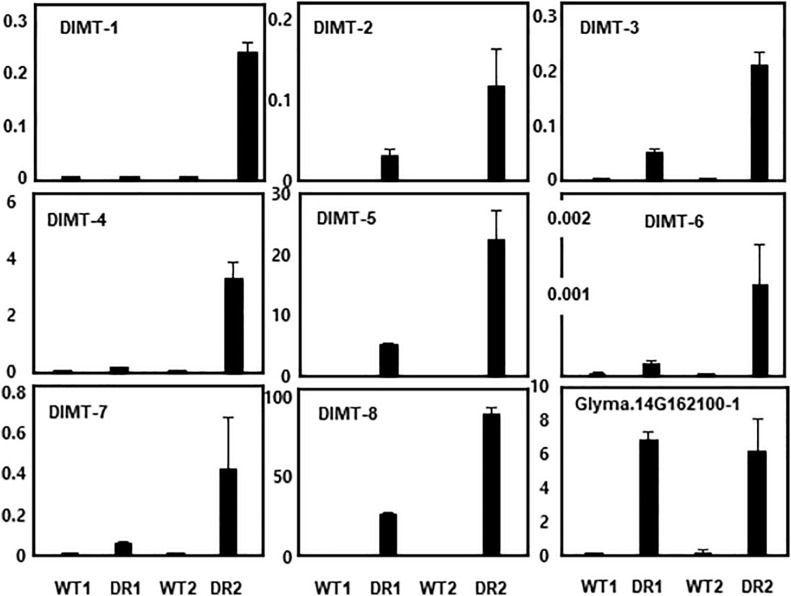
qRT-PCR analysis of selected drought stress memory genes (DIMTs). Eight DIMTs were selected and their expression levels were determined by qRT-PCR. DIMT-1, Glyma.11G025600-1; DIMT-2, Glyma.06G061900-1; DIMT-3, Glyma.U018200-1; DIMT-4, Glyma.04G042300-1; DIMT-5, Glyma.12G149100-1; DIMT-6, Glyma.12G116800-1, DIMT-7, Glyma.20G155100-1; DIMT-8, Glyma.03G144400-1. Transcript levels of a drought inducible gene (Glyma.14G162100-1) were used as a negative control (non-stress memory gene).

The DIMT genes we tested included those containing domains encoding thaumatin (osmotin), WRKY, SMP (responsive ABA), MYB, NAM (NAC), PP2C, AP2 (DREB-responsive element), and LEA. These genes were also highly induced in the secondary drought stress conditions (Figure 3), confirming that the overall qRT-PCR data were consistent with the microarray data. In this experiment, the expression pattern of a PP2C gene (Glyma.14G162100-1) homologous to Arabidopsis *HAI1* was tested as a negative control. Transcript levels of this gene in the first and second stage of drought stress were comparable to each other. Thus, this gene is not a stress memory gene, but a simple drought-responsive gene.

## Discussion

We identified 392 DIMTs and 613 DRMTs in soybean through microarray analysis (Supplementary Table 1B). The DIMTs exhibiting significantly elevated transcript levels were involved in tolerance responses to abiotic stresses. The DIMTs include those encoding transcription factors, a trehalose biosynthesis enzyme, LEA proteins, and PP2C family proteins. Trehalose is a non-reducing disaccharide that has high water-holding activity and thereby maintains membrane fluidity under low-water conditions (Iordachescu and Imai, 2008; Delorge et al., 2014). LEA proteins are extremely hydrophilic; thus, the accumulation of these proteins gives plants the desiccation tolerance necessary for dealing with water-deficit stress (Hundertmark and Hincha, 2008; Hand et al., 2011). PP2Cs negatively regulate ABA-activated signaling by counteracting protein kinases, allowing plant cells to maintain the phosphorylation balance needed to control ABA signaling processes (Ma et al., 2009; Umezawa et al., 2009). By contrast, DRMTs exhibiting highly reduced levels of transcripts under second drought conditions include those involved in photosynthesis and primary metabolism.

However, it appears that gene sequence homology or protein structure does not determine whether the gene displays a memory function or is simply a drought-responsive gene. For example, among the 31 drought-induced transcripts encoding PP2C family proteins, only 10 exhibited expression patterns indicative of memory function (Table 1). As confirmed in a qPCR experiment (Figure 3), transcript levels of the PP2C gene (Glyma.14G162100-1) homologous to Arabidopsis *HAI1* in the first and second stage of drought stress were comparable to each other. Thus, this gene is not a stress memory gene; rather, it is a simple drought-responsive gene. Supporting this observation, the Arabidopsis gene *RD29B* displayed a memory function, whereas its homologous copy *RD29A* was non-trainable during repeated dry stress (Ding et al., 2012). Revealing the molecular mechanisms of transcriptional memory responses is critical for defining the characteristics of stress memory genes.

The soybean drought-induced stress memory genes identified in this study included those involved in the dehydration memory responses of Arabidopsis (Ding et al., 2013). In Arabidopsis, genes implicated in responses to salt, salinity, cold/heat acclimation, and ABA constitute approximately one quarter of the drought-induced memory genes, in addition to LEA genes (Ding et al., 2013). Genes of chloroplast and thylakoid membrane-associated functions comprised the dehydration-repressed memory genes. Co-expression networks revealed that the soybean drought memory genes were equivalent to Arabidopsis genes having similar functions. Thus, dehydration stress memory in both dicot plants implies reinforcement of the cellular metabolic processes needed for adaptive and protective functions and repression of photosynthesis.

However, studies of other crop plants identified drought memory genes that function in species-specific ways, as well as genes that function similarly. *Z. mays* exhibited a conserved pattern of memory responses to dehydration stress, as also observed in Arabidopsis (Ding et al., 2014). However, comparison of the cellular functions encoded by the memory genes in the two species revealed remarkable differences. *Z. mays* proteins associated with only four chloroplast and two thylakoid membrane functions were encoded by drought-repressed memory genes, in contrast with the 128 Arabidopsis drought-repressed memory genes implicated in these functions. *Z. mays* genes implicated in abiotic stress responses were also represented by much lower numbers of drought-repressed memory genes than in Arabidopsis.

In the case of potato, drought was memorized by genes involved in photosynthesis, signal transduction, sugar metabolism, flavonoid metabolism, and the biosynthesis of protease and protease inhibitors, transporters, and transcription factors (Chen et al., 2020). The expression levels of most photosynthesis-related genes during the second drought were higher than during the first drought. In addition, Li et al. (2019) identified clusters of rice stress memory gene transcripts involved in ABA signaling, the production of protective substances, and processes such as photosynthesis and DNA methylation. Interestingly, most memory transcripts associated with photosynthesis were sharply reduced by the first drought treatment, and then maintained at a stable level with the subsequent drought treatments. Thus, it appears that photosynthesis efficiency in rice plants was decreased by the initial drought stress, but recovered and improved during the subsequent treatments.

Wide exploitation of drought stress memory to improve crop yield has been hampered by the fact that stress-induced memories are relatively short term (Kinoshita and Seki, 2014; Avramova, 2015). In Arabidopsis, dehydration stress-induced transcriptional memory persists for 5 days under watered conditions (Ding et al., 2012). In most cases, the duration of a somatic memory is limited to one generation (Lämke and Bäurle, 2017). The acquired stress memory may be reset during recovery, subsequent growth, and meiosis, as observed in mammalians (Crisp et al., 2016). How plants overcome such resetting processes during meiosis, to transmit the stress memory to the next generation, remains to be intensively investigated.

The drought stress memory genes identified in this study will facilitate research on the epigenetic mechanisms and biotechnological applications of drought stress memory. Studies of the chromatin architecture of the gene loci should make it possible to understand the nature of the memory response. In addition, the memory genes could be used as markers to assess the transmission of an acquired memory across generations in soybean, and to develop proper priming techniques (method, strength, frequency, interval, etc.) to prolong the memory in the next generation or even farther.

## Data Availability Statement

The data can be accessed using the accession number GSE153660 on NCBI.

## Author Contributions

Y-KK designed the DNA chip and conducted the bioinformatics analyses. SC, N-IO, and NN performed the experiments. J-JC designed the whole research scheme and interpreted the data. Y-KK and J-JC wrote the manuscript. J-JC edited the manuscript. All the authors contributed to the article and approved the submitted version.

## Conflict of Interest

The authors declare that the research was conducted in the absence of any commercial or financial relationships that could be construed as a potential conflict of interest.

## Footnotes

1 http://www.phytozome.net

2 http://www.agilent.com

3 http://www.bioconductor.org/

4 http://master.bioconductor.org/

5 http://bioinfo.mju.ac.kr/arraynet/

6 http://www.ncbi.nlm.nih.gov/geo/

7 www.phytozome.net

8 http://phytozome.jgi.doe.gov/
