# Supplementary material for: Recurrent Drought Conditions Enhance the Induction of Drought Stress Memory Genes in Glycine max L
Source: Front Genet. 2020 Oct 9;11:576086. doi: 10.3389/fgene.2020.576086 (PMC7581891; doi:10.3389/fgene.2020.576086)
Supplement: Supplementary file 1 [file Data_Sheet_1.PDF]

Frontiers in Genetics

Recurrent drought conditions enhance the induction of drought stress memory genes in *Glycine max* L.

*Yeon-Ki Kim, Songhwa Chae, Nam-Iee Oh, Nguyen Hoai Nguyen, and Jong-Joo Cheong**

Supplementary Figure 1.
Drought treatment of soybean seedlings.

Supplementary Figure 2.
Experimental design for identification of drought memory genes.

Supplementary Figure 3.
Normalization of microarray data.

Supplementary Figure 4.
Gene Ontology (GO) terms.

Supplementary Figure 5.
Selected drought stress memory genes (DIMTs) analyzed by qRT-PCR.

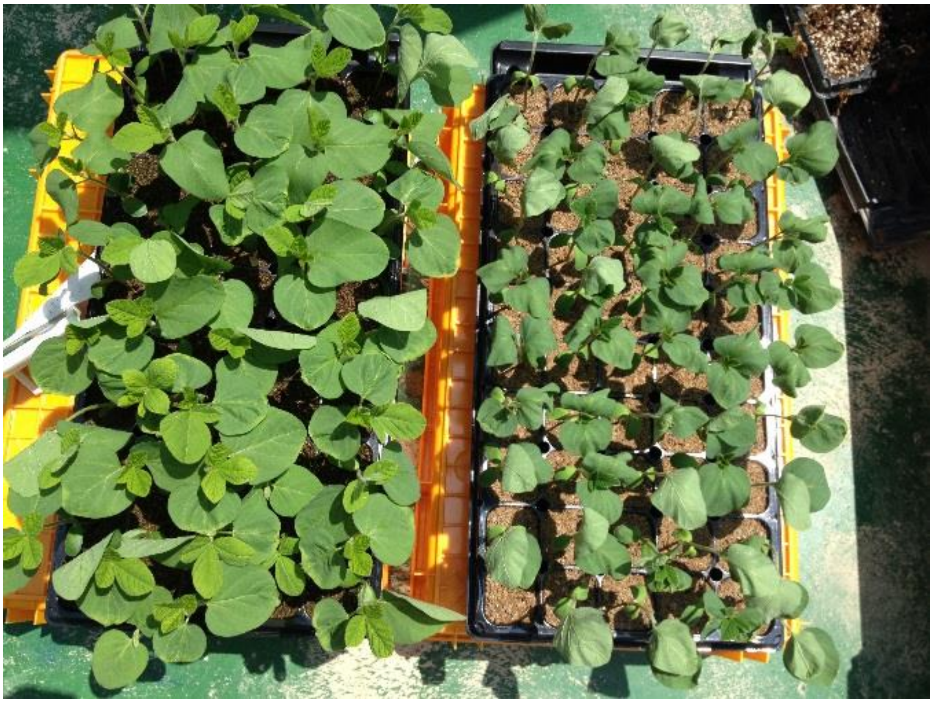

WT1 **DR1**

Supplementary Figure 1. Drought treatment of soybean seedlings. Soybean seeds were sown on potting soil at 1.5-cm depth in a 50-well plate with one seed per well (35 mm W × 35 mm L × 45 mm D), and grown in a growth chamber at 28°C and 50–60% relative humidity under a 16-h light (8,000 lux)/8-h dark photoperiod. Water was supplied from the watering tray located beneath the plate. The seedlings were grown for 7 d, then supplied with water (WT1) or exposed to drought stress by removal of the water tray (DR1) for 4 d.

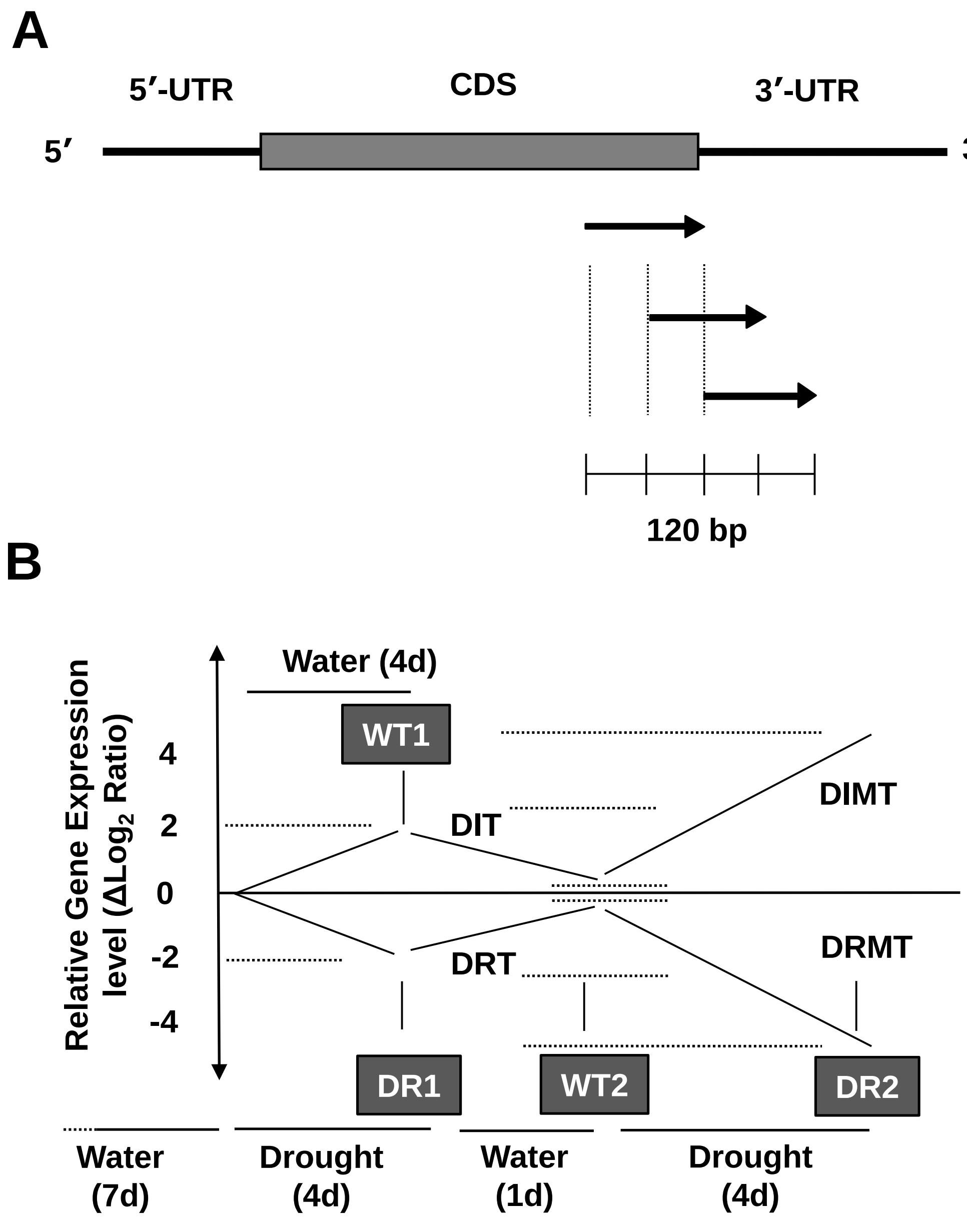

Supplementary Figure 2. Experimental design for identification of drought memory genes. (A) Probe design for soybean chromosomal genes. Three 60-nt feature probes were designed from a representative transcript of each gene, beginning 60 bp upstream from the end of the stop codon and shifting downstream at 30-bp intervals. (B) Sampling of drought-treated soybean leaves. Soybean seedlings were grown for 7 d, then exposed to drought stress by removal of the water tray for 4 d. From the normalized microarray data, the primary drought-responsive transcripts (induced or repressed at the first drought treatment) were determined. DIT, drought-induced transcript; DRT, drought-repressed transcript; DIMT, drought memory-increased transcript, DRMT, drought memory-decreased transcript.

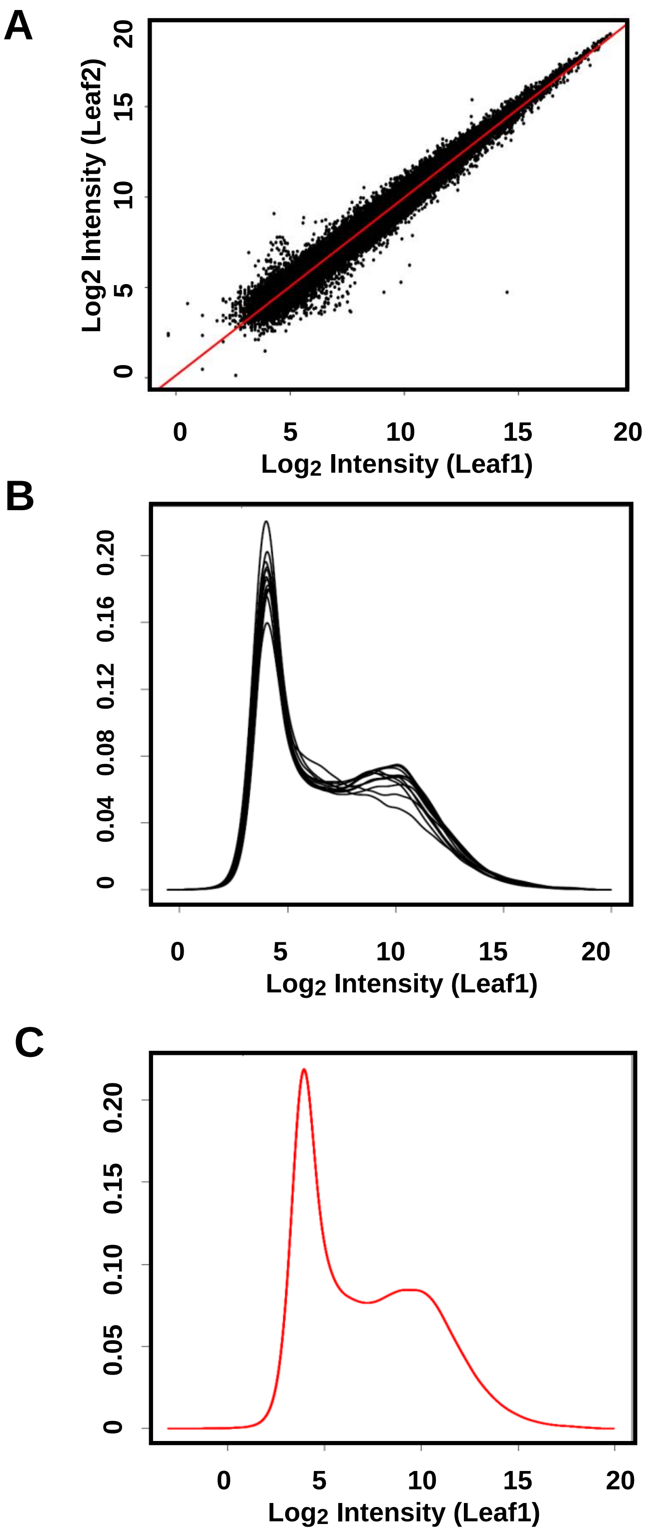

Supplementary Figure 3. Normalization of microarray data. The data were processed using the Limma package in the R computing environment (www.bioconductor.org/). The distribution of probe intensities from eight microarray datasets was analyzed using the plotDensities function. Frequency densities before and after normalization were plotted against log2-based intensities. (A) Consistency between microarrays. Log2-based intensities of two WT1 samples were compared. Linear model: $y = 0.98x + 0.14$. Pearson correlation coefficient: 0.97. (B) Signal distribution after background correction. (C) Normalization between slides.

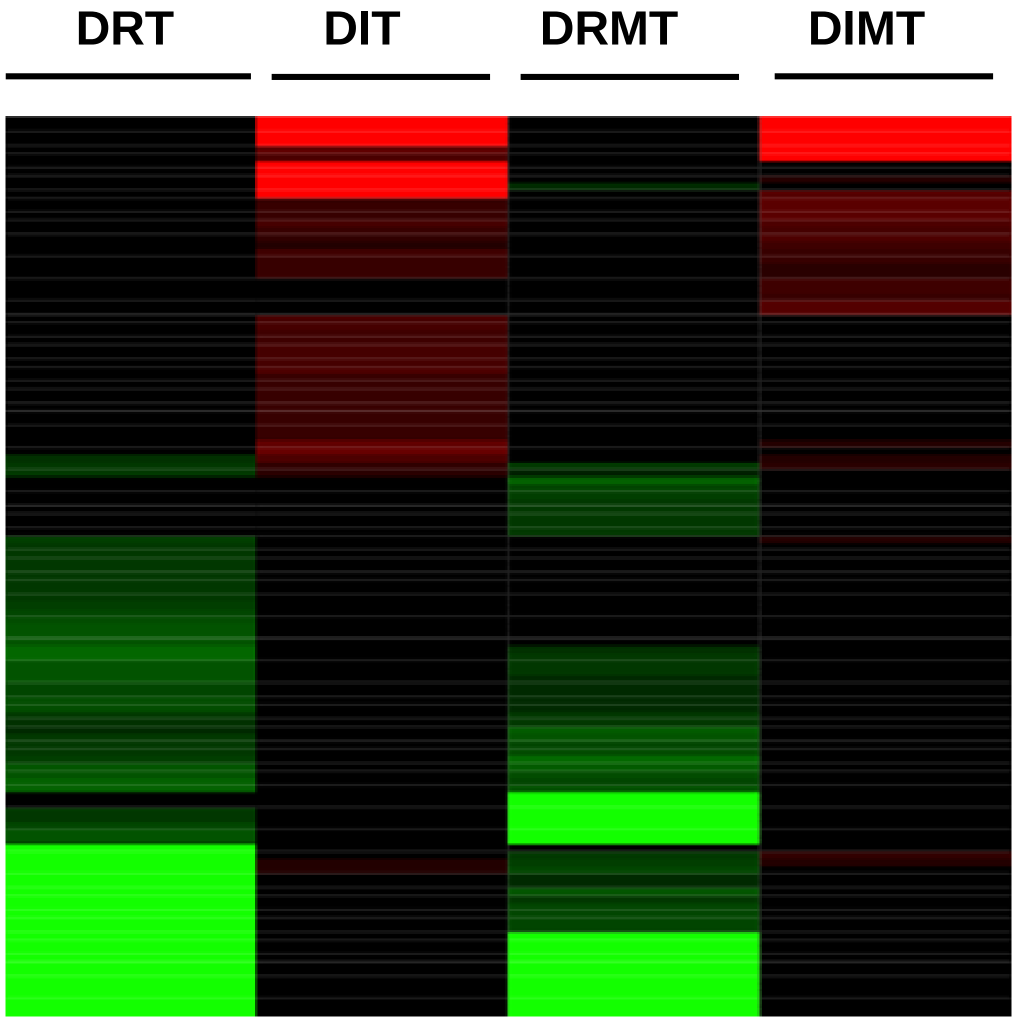

Supplementary Figure 4. Gene Ontology (GO) terms. Compared with transcription levels in well-watered soybean plants, genes that decreased (DRT) or increased (DIT) 4-fold after the first drought stress treatment were identified. To find the genes involved in the drought memory process, the plants were re-watered and then water deprived a second time (second stress). GO terms of genes that decreased (DRMT) or increased (DIMT) 4-fold, when compared to those observed under the first drought stress, were considered to be drought stress memory terms.

SMG	SEQ_ID (Glyma.)	Log2[Fold Change]				Domain
		DR1/WT1	WT2/WT1	DR2/WT1	DR2/DR1	
DIMT-1	11G025600-1	2.069	0.219	8.774	6.705	PF00314.15 Thaumatin
DIMT-2	06G061900-1	3.166	0.383	6.307	3.141	PF03106.13 WRKY
DIMT-3	U018200-1	3.775	0.563	8.066	4.291	PF04927.10 SMP
DIMT-4	04G042300-1	2.965	0.480	8.948	5.983	PF00249.29 MYB
DIMT-5	12G149100-1	6.320	1.141	10.568	4.248	PF02365.13 NAM
DIMT-6	12G116800-1	3.264	1.434	6.430	3.166	PF00481.19 PP2C
DIMT-7	20G155100-1	3.629	0.597	6.123	2.494	PF00847.18 AP2
DIMT-8	03G144400-1	10.630	4.436	12.657	2.027	PF03760.13 LEA_1
	14G162100-1	6.808	2.629	7.211	0.430	PF00481.19 PP2C

Genes	primer	Sequence (from 5'-3')	PCR product size (bp)
DMIT-1	FW	TTTGAAGGTCAGATTTATGTGCG	70
	RV	AACCAGTGCTGGATTGCTACA	
DMIT-2	FW	TGATTACCGAAACACTACTTGGA	136
	RV	CCTATTGGAAGGACTGAGGCA	
DMIT-3	FW	AAATGACGCCGATGCGATTG	121
	RV	TCGCATGACGTGACTGTTGA	
DMIT-4	FW	TGCAAACCGATGCCATTAGG	139
	RV	ATCACTATTTCTCTGCGATTTCACC	
DMIT-5	FW	GGGTCAGCAAGTTGAGTTCG	140
	RV	TGTGTCTAGCCGAAAAGAATGAC	
DMIT-6	FW	GGCAAGTCACGTGCTCAAAA	200
	RV	TGGTGTTCCAAACCCTGCTT	
DMIT-7	FW	TGGGACAAGTAGAATAGTGTCCG	70
	RV	TTTGGACACCTAAAATGAGCAACC	
DMIT-8	FW	AACCTAAGTGAGAAACAAAGAAAGG	136
	RV	GCCTTGGTTTTCTCCATGCC	
Glyma. 14G162100-1	FW	ACTTGCCAGACGAAGATCCG	76
	RV	CAGAATCCACCGCCAGAAGT	
60S RNA (standard)	FW	AAAGTGGACCAAGGCATATCGTCG	125
	RV	TCAGGACATTCTCCGCAAGATTCC	

Supplementary Figure 5. Selected drought stress memory genes (DIMTs) analyzed by qRT-PCR. Eight DIMTs were selected and their expression levels were determined by qRT-PCR. Transcript levels of a drought inducible gene (DIT-1) were used as a negative control (non-stress memory gene)..Primers used for qRT-PCR in this study. FW, forward primer sequence; RV, reverse primer sequence.
